# Assessment of I-125 seed implant accuracy when using the live-planning technique for low dose rate prostate brachytherapy

**DOI:** 10.1186/1748-717X-7-196

**Published:** 2012-11-22

**Authors:** Joshua Moorrees, John M Lawson, Loredana G Marcu

**Affiliations:** 1 Department of Medical Physics, Royal Adelaide Hospital, Adelaide, North Terrace, SA, 5000, Australia; 2 Faculty of Science, University of Oradea, Oradea, 410087, Romania; 3 School of Chemistry and Physics, University of Adelaide, North Terrace, Adelaide, SA, 5000, Australia

**Keywords:** Prostate, Brachytherapy, I-125, Dosimetry, Real-time planning

## Abstract

**Background:**

Low risk prostate cancers are commonly treated with low dose rate (LDR) brachytherapy involving I-125 seeds. The implementation of a ‘live-planning’ technique at the Royal Adelaide Hospital (RAH) in 2007 enabled the completion of the whole procedure (i.e. scanning, planning and implant) in one sitting. ‘Live-planning’ has the advantage of a more reliable delivery of the planned treatment compared to the ‘traditional pre-plan’ technique (where patient is scanned and planned in the weeks prior to implant). During live planning, the actual implanted needle positions are updated real-time on the treatment planning system and the dosimetry is automatically recalculated. The aim of this investigation was to assess the differences and clinical relevance between the planned dosimetry and the updated real-time implant dosimetry.

**Methods:**

A number of 162 patients were included in this dosimetric study. A paired *t*-test was performed on the D90, V100, V150 and V200 target parameters and the differences between the planned and implanted dose distributions were analysed. Similarly, dosimetric differences for the organs at risk (OAR) were also evaluated.

**Results:**

Small differences between the primary dosimetric parameters for the target were found. Still, the incidence of hotspots was increased with approximately 20% for V200. Statistically significant increases were observed in the doses delivered to the OAR between the planned and implanted data; however, these increases were consistently below 3% thus probably without clinical consequences.

**Conclusions:**

The current study assessed the accuracy of prostate implants with I-125 seeds when compared to initial plans. The results confirmed the precision of the implant technique which RAH has in place. Nevertheless, geographical misses, anatomical restrictions and needle displacements during implant can have repercussions for centres without live-planning option if dosimetric changes are not taken into consideration.

## Background

A common treatment option for low grade prostate cancers is low dose rate (LDR) seed brachytherapy. It is usually employed as a monotherapy. Early developments of this treatment technique would implant the seeds into the prostate via retropubic surgery. This was later improved so that the seeds were implanted via the perineum without the need for surgery [1]. This procedure is done by placing the seeds into large gauge needles which are then inserted to the required depth before the seeds are deployed as the needle is withdrawn. To guide the placement of these seeds some form of image guidance is typically employed. The most commonly used is a combination of ultrasound (US) and fluoroscopy, where the ultrasound imaging is achieved via a trans-rectal ultrasound (TRUS) probe.

LDR seed brachytherapy has been performed at the Royal Adelaide Hospital (RAH) since September 2004. Initially the two step treatment technique was employed where the patient had a TRUS volume study on a separate day to the implant. The TRUS scan enabled the assessment of prostate volume (prostate volumes ranging from 15 cc to 50 cc were deemed eligible) and provided images for contouring and treatment planning. The time gap between the TRUS scan and the seed implant was usually 4 weeks. On the morning, prior to the implant the needles were manually loaded with the planned seed configurations. These needles were then implanted after aligning the planning US images of the prostate with the live US images of the prostate as seen on the implant day.

However, the time gap between the TRUS scan and the implant procedure was shown to lead to suboptimal consequences due to difficulties in reproducing the patient alignment. Often it was quite difficult to align the planning US images with the treatment images. This challenge was possibly caused by several factors, such as: (1) changes in patient’s anatomy between the two scans; (2) changes in patient positioning on the operating couch. Thus the delivered seed dosimetry was likely to be significantly different from the initially planned one [2]. To eliminate this problem and, at the same time, to optimise treatment flow, the quality of the implant and hence treatment outcome, it was decided to move to a one step live planning technique. In a one step live planning technique the TRUS scan and implant are performed on the same day, significantly reducing the chance for movement and the changes in prostate position between pre-implant TRUS and live-implant.

The treatment procedure on the day is as follows: First the patient in anaesthetised, catheterised and placed into stirrups. Next, an ultrasound scan (BK Medical Flex Focus 1202–400 US mated with Nucletron SPOT PRO™ Version 3.1 TPS) is taken of the prostate upon which the prostate, PTV and OAR are contoured by both the urologist and radiation oncologist in consultation with each other. In most cases the urethra is both identified and contoured through the use of a contrast medium (aerated jelly) which is injected into the catheter. The visualised catheter (5 mm diameter) is then taken as being representative of the urethral volume. It should be noted that patients who are eligible to be treated via LDR seeds are generally not given androgen deprivation therapy (ADT) prior to the treatment day. A small number (~5%) are given ADT in order to bring the prostate down to a more manageable size. Literature suggests that shrinkage of around 30% is achieved through the use of ADT and the maximum shrinkage occurs after a period of three months [3].

Once this process is complete, the planner (medical physicist or radiation therapist) will create a plan for the distribution of seeds within the prostate using the Nucletron SPOT Pro system and a modified uniform loading of seeds [4,5]. After treatment plan optimisation and approval, pre-loaded needles (Oncura Rapid Strand RX) are inserted by the urologist. The seeds are then deployed to their respective positions as indicated by the plan under fluoroscopic and ultrasound guidance. Anchor needles (or fixation needles) are not used at our centre. However all needles of the same retraction distance (relative to the prostate base plane) are inserted and then aligned (with US and fluoroscopy guidance) prior to seed deployment. At each retraction the prostate base plane is checked and amendments made if necessary again prior to seed deployment. After the completion of the planned implant the radiation oncologist will inspect the plan on the TPS (using the dosimetry from the live updates of the actual implanted seed positions), plus check the ultrasound and fluoroscopic images, to determine if the implant dose coverage is satisfactory. If there were any gaps in the dose coverage of the target the radiation oncologist may request more seeds to be implanted at a given location within the prostate by the urologist.

The final quality of the implant is assessed via post implant dosimetry. In our centre, post implant dosimetry is completed based on US-CT image co-registration, with the pelvic CT scan taken on day 0 or 1 after the implant. Details on post implant dosimetry technique and results have been recently published [6]. According to the latest ABS guidelines [7] the post implant dosimetry (PID) is considered to be a mandatory component of the implant’s quality assurance and should be undertaken within 60 days of the implant. While CT-MR fusion-based PID is recommended, CT-based PID is also considered acceptable.

It should be noted that prior to the treatment day the medical physicist will have performed an independent assay of the seeds used to ensure the correct activity is used by the TPS. Initially for this technique the needles were manually pre-loaded on site prior to implant, but later we moved to purchasing manufacturer pre-loaded needles. In each case a set of generic seed-needle configurations were determined from analysing the seed configurations used for previous implants. The large number of seeds ordered per patient (110 seeds) allow ample seed configuration combinations to be pre-loaded.

The SPOT Pro planning software used at the RAH allows the user to update the location of the implanted needles in real-time on the treatment planning system and hence automatically recalculate the plan dosimetry, to accurately reflect what was actually implanted. This is important, as the theoretically planned grid positions might not always be practically attainable. There are a few factors which can possibly impede the perfect reproduction of the treatment plan:

*Operator-related*: needles can miss the planned grid position by a few millimetres, so they do not exactly end up where the implanting physician intended them to go. Though the error is usually small, overall it contributes to the difference between the planned and implanted dosimetry.

*Patient anatomy-related*: there are situations when the physician may need to implant at another grid position than the planned one due to interference such as pubic arch. Calcifications inside the prostate gland could also lead to similar interferences necessitating slight adjustments in needle positions.

*Prostate gland-related*: small changes in the prostate glad volume during implant due to swelling (from oedema) might influence the decision of needle placement.

These offsets (both small and large) could potentially add up to give a significantly different dosimetry to that which was initially planned and approved.

The current work will identify any significant differences for the main dosimetric parameters used in the reporting of LDR prostate treatments when comparing the planned dosimetry and the final updated real-time implant dosimetry.

## Methods

This report analysed the dosimetry data for all patients treated since the inception of the ‘live planning’ technique at the RAH, which amounted to 162 patients. Given that the LDR seed implant programme at RAH started in 2004 and that the 2 step live planning procedure with manufacture pre-loaded needles did not start until Nov 2007, it was felt that during the studied period all staff involved in prostate brachytherapy were experienced in the procedure. As such, this data should be considered to be representative of a well established program. The prescribed dose to the target volume is 145 Gy and the average activity per seed is 0.395mCi, with an average of 70 seeds being implanted per patient.

**Figure 1:**
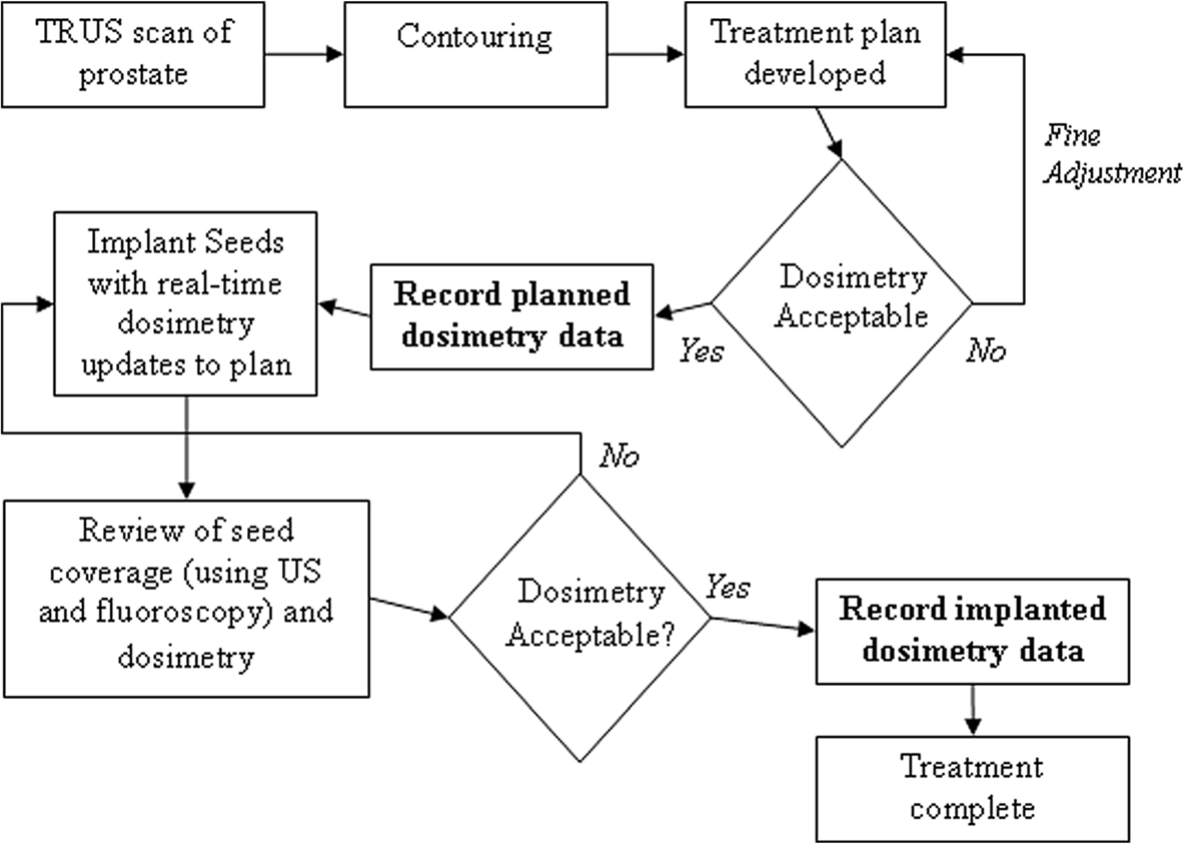
LDR brachytherapy implant workflow with one step live planning technique (highlighted in bold are the two dosimetry data recording points).

The LDR brachytherapy implant workflow during this period is represented in Figure 1. Of note here are the two dosimetry data recording points, i.e. (1) prior to implantation and (2) after the completion of the implant and final plan analysis review. These are the two points at which data was collected and analysed for this study. The first will be termed the ‘planned’ dosimetry and the second the ‘implanted’ dosimetry.

Contour delineation for the prostate target and organs at risk was performed on the TRUS transverse images, where the prostate is defined as the clinical target volume (CTV); the PTV includes the CTV plus treatment margins of 3–5 mm (depending on the patient’s pathology); and the OAR include the prostatic urethra and anterior rectal wall. For the prostate and PTV the dosimetric parameters analysed were the following: (1) the dose received by 90% of the relative volume (D90), (2) the relative volume to receive 100% of reference dose (V100), (3) the relative volume to receive 150% of the reference dose (V150) and (4) the relative volume to receive 200% of the reference dose (V200). These parameters offer both a good representation of the prostate and PTV coverage (via D90 and V100) and the incidence of ‘hot spots’ with V200 and V150. Based on recommendations for reporting from GEC-ESTRO the dosimetric parameters analysed for urethra were D10 and D30 while for rectum were the dose received by a volume of 2cc of the prostate (D2cc) and the dose received by a very small volume (D0.1cc) [8].

To determine if there is any statistically significant difference between the planned and implanted dosimetric parameters a paired *t* test was performed on the two samples. The mean percent difference between the two data sets was also determined along with the standard deviation of these differences.

## Results and discussion

The results obtained using the methodology outlined previously were divided into two sections: one for the treated volumes, i.e. prostate and PTV, and the other for the OAR. It should be noted that these mean difference and standard deviation are in terms of percent difference relative to the planned dosimetry.

### Prostate and PTV

First the average values and standard deviations for each of the dosimetry parameters can be seen in Table 1 for both the planned and implanted plans. This data shows an increase in all three parameters although this increase is negligible for V100.

**Table 1:** Mean values for D90, V100, V150 and V200 for the prostate along with the associated standard deviations

**Dosimetry Parameter**	**Planned**	**St Dev**	**Implanted**	**St Dev**
**D90 (Gy)**	175.5	9.6	177.7	10.5
**V100 (%)**	97.9	1.6	97.9	1.5
**V150 (%)**	64.1	7.3	66.5	7.9
**V200 (%)**	21.6	5.9	26.1	7.3

Table 2 shows the mean for the differences between the planned and implanted dosimetry data shown in Table 1 as well as the statistical data from the paired *t* test.

**Table 2:** Percent differences and statistics for target data

**Dosimetry Parameter**	**Mean % Diff**	***t *test score**	**P value**
**D90**	1.24	2.87	0.0047
**V100**	0.09	1.00	0.3209
**V150**	3.81	5.70	0.0001
**V200**	20.81	10.95	0.0001

A slightly significant difference in D90 between the planned and implanted dose distribution is observed, however the most significant difference for the target itself is in V150 and V200. For these parameters there is approximately a 4% and 20% average increase in the size of the hot spots respectively, which as reflected in the p values, is very significant.

**Figure 2:**
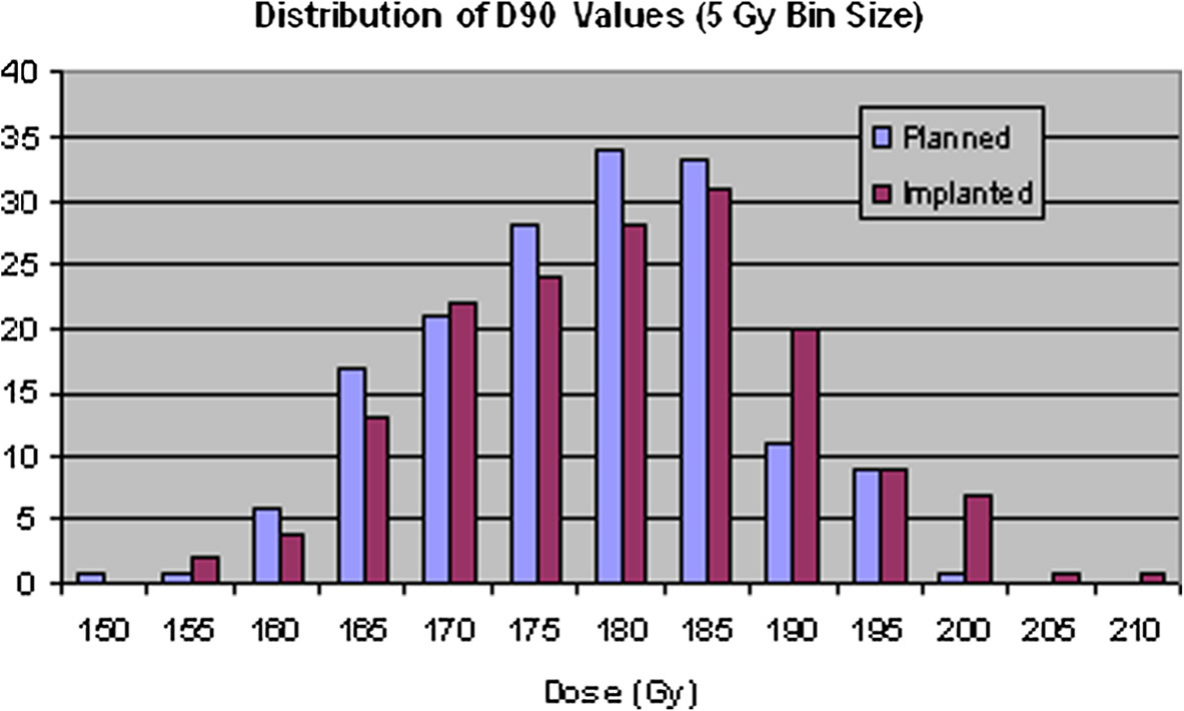
Distribution of prostate D90 values for planned and implanted dosimetry.

**Figure 3:**
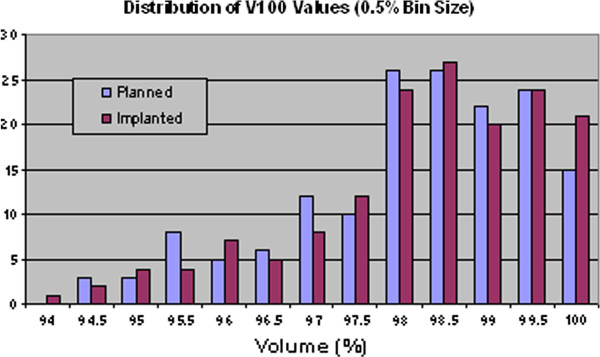
Distribution of prostate V100 values for planned and implanted dosimetry.

**Figure 4:**
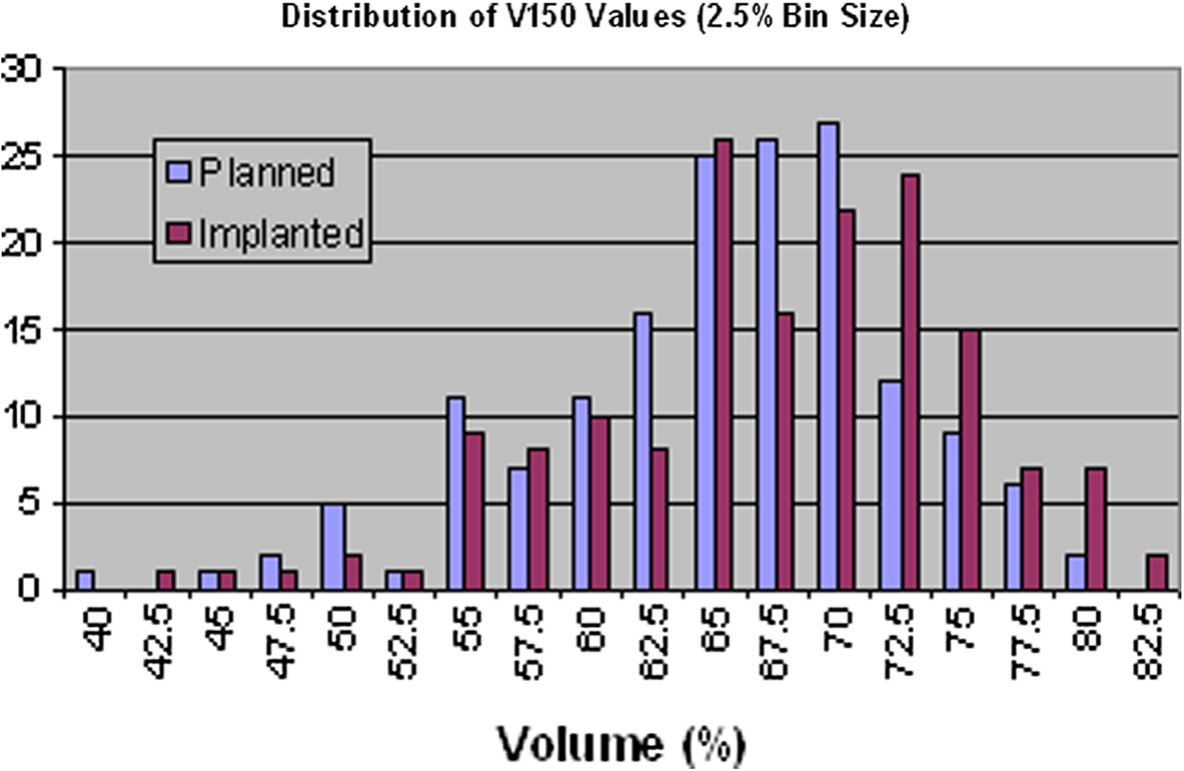
Distribution of prostate V150 values for planned and implanted dosimetry.

**Figure 5:**
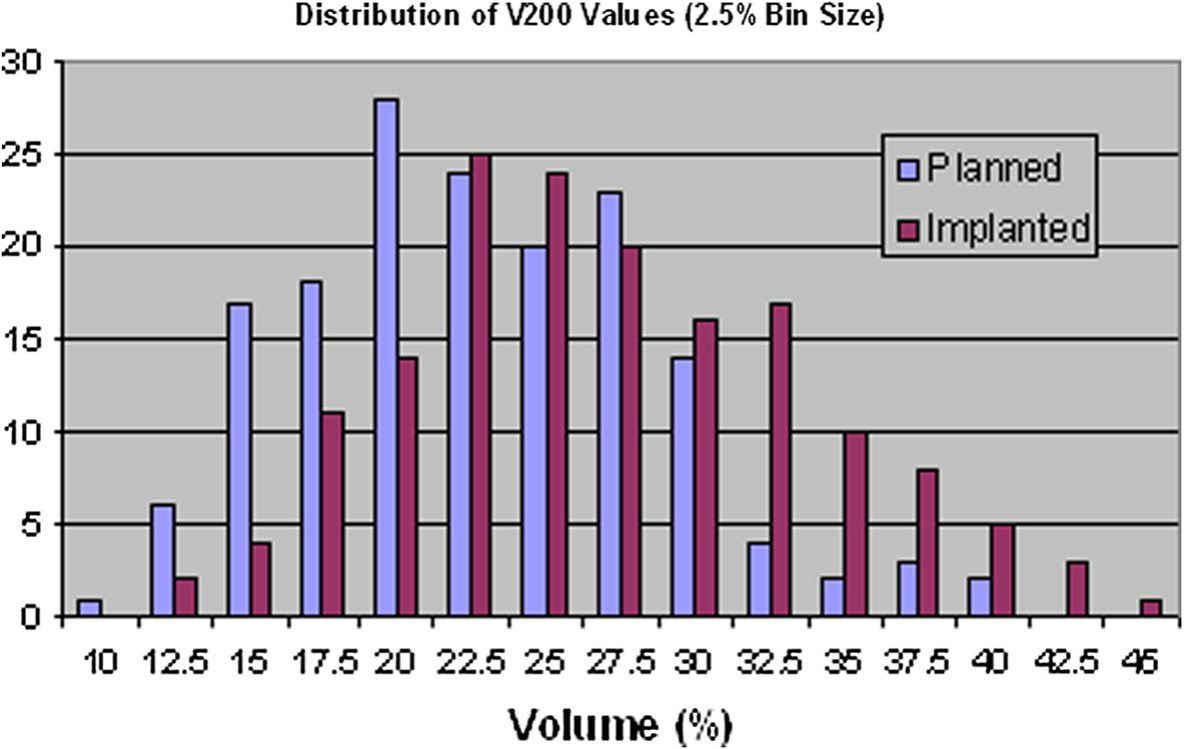
Distribution of prostate V200 values for planned and implanted dosimetry.

By binning the prostate volume data, it is possible to show the distributions of the dosimetry parameters graphically, as illustrated in Figure 2, Figure 3, Figure 4 and Figure 5. Visually, this supports the statistical evidence as a clear increase in the distribution of D90, V150 and V200 while there is no clear change in the data for V100.

Given that the prostate, as a volume, is a subset of the PTV it would be expected for the dosimetry results to be reasonably similar and this is what is shown in Tables 3 and Table 4. While there is no significant difference in the D90 values, there is a significant increase in V150 and V200 of 4% and 20% respectively, the same order as for the target.

**Table 3:** Mean values for D90, V100, V150 and V200 for the PTV along with the associated standard deviations

**Dosimetry Parameter**	**Planned**	**St Dev**	**Implanted**	**St Dev**
**D90 (Gy)**	168.0	8.0	169.1	10.3
**V100 (%)**	96.6	1.7	96.6	1.8
**V150 (%)**	60.2	6.7	62.4	7.4
**V200 (%)**	20.0	5.1	24.0	6.3

**Table 4:** Percent differences and statistics for PTV data

**Dosimetry Parameter**	**Mean % Diff**	***t *test score**	**P value**
**D90**	0.61	1.74	0.0842
**V100**	0.07	0.68	0.4990
**V150**	3.67	4.95	0.0001
**V200**	20.47	11.30	0.0001

### Organs at risk

As with the prostate and PTV there is a general increase in the value of the dosimetry parameters used for the urethra in LDR prostate treatments from the planned to the implanted treatment. This increase can be seen in Table 5 while the statistical data in Table 6 illustrates that these are significant changes. It should be noted however, that the changes are reasonably small in the actual values, of only around 3%.

**Table 5:** Mean values for D10 and D30 for the urethra along with the associated standard deviations

**Dosimetry Parameter**	**Planned**	**St Dev**	**Implanted**	**St Dev**
**D10 (Gy)**	190.6	11.2	196.2	17.1
**D30 (Gy)**	183.3	10.9	188.2	14.2

**Table 6:** Percent differences and statistics for urethra data

**Dosimetry Parameter**	**Mean % Diff**	***t *test score**	**P value**
**D10**	2.90	5.06	0.0001
**D30**	2.64	5.45	0.0001

**Figure 6:**
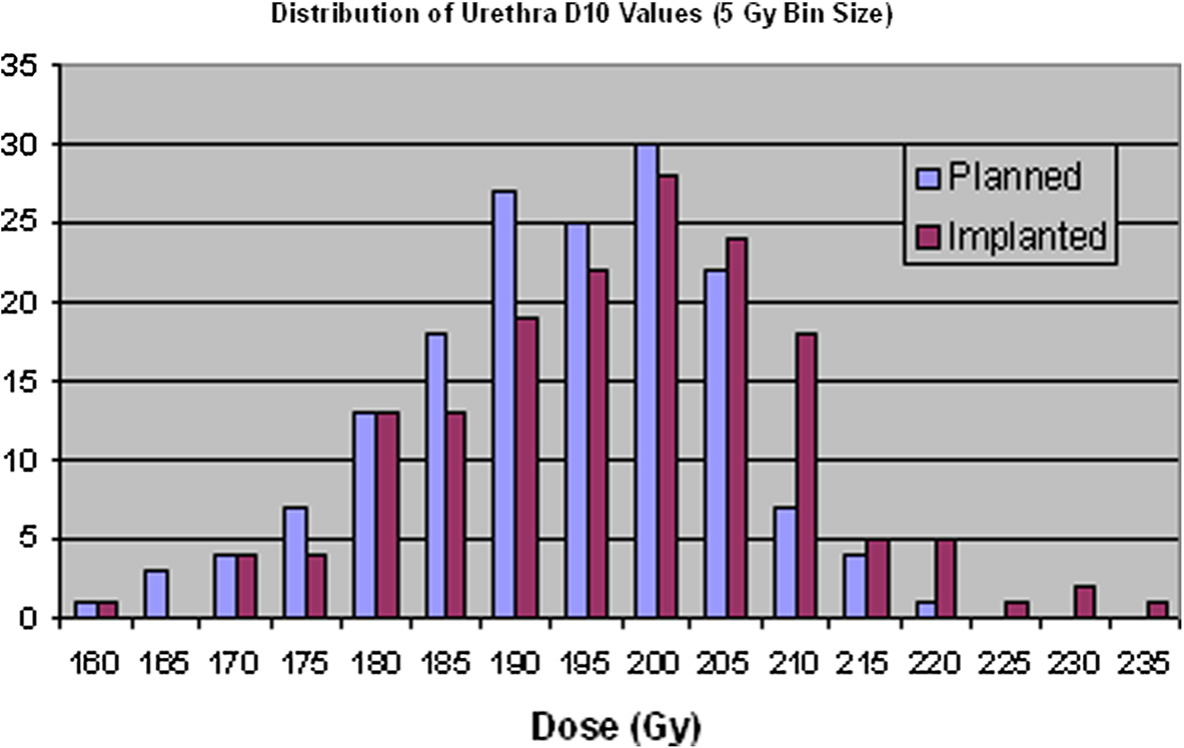
Distribution of urethra D10 values for planned and implanted dosimetry.

**Figure 7:**
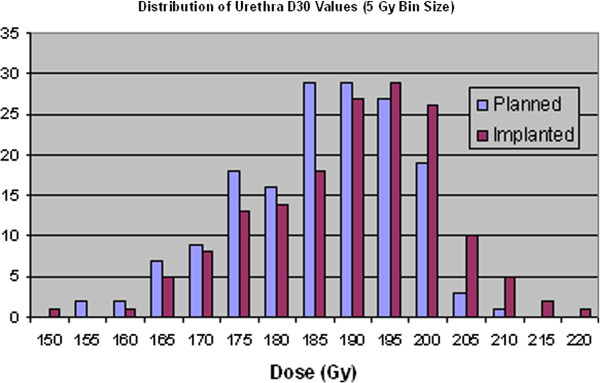
Distribution of urethra D30values for planned and implanted dosimetry.

Figure 6 and Figure 7 clearly illustrate this shifting of urethral doses to higher levels for the implanted seed distribution compared to the planned seed distribution.

**Figure 8:**
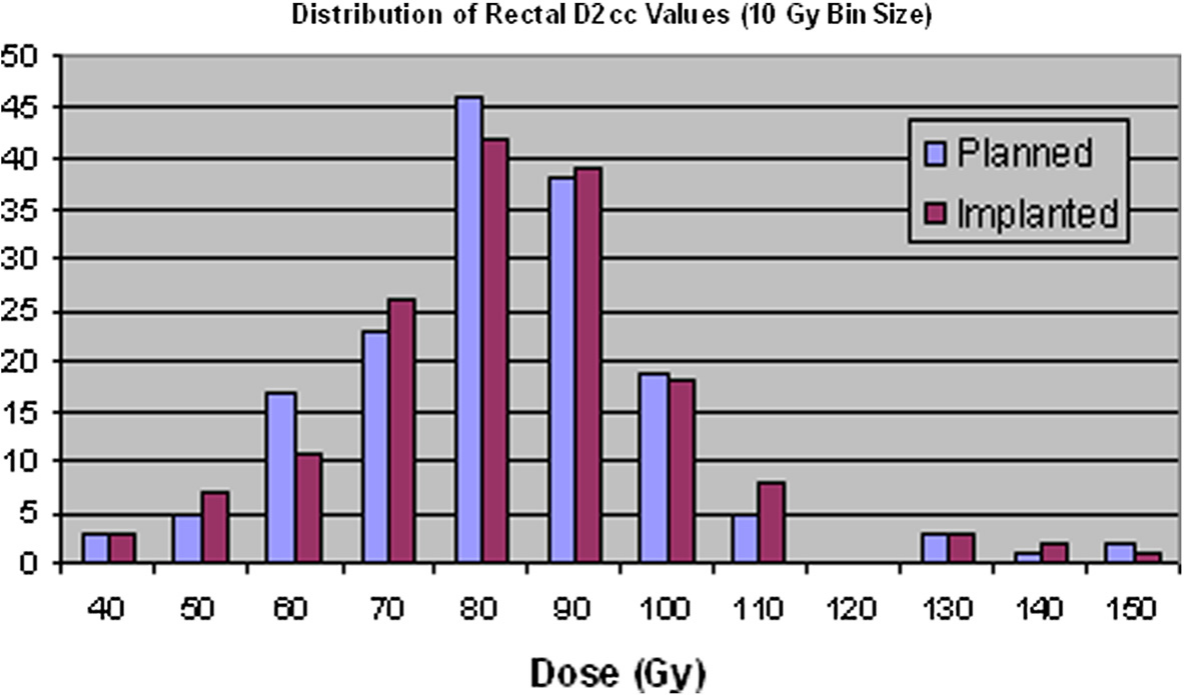
Distribution of rectal D2cc values for planned and implanted dosimetry.

**Figure 9:**
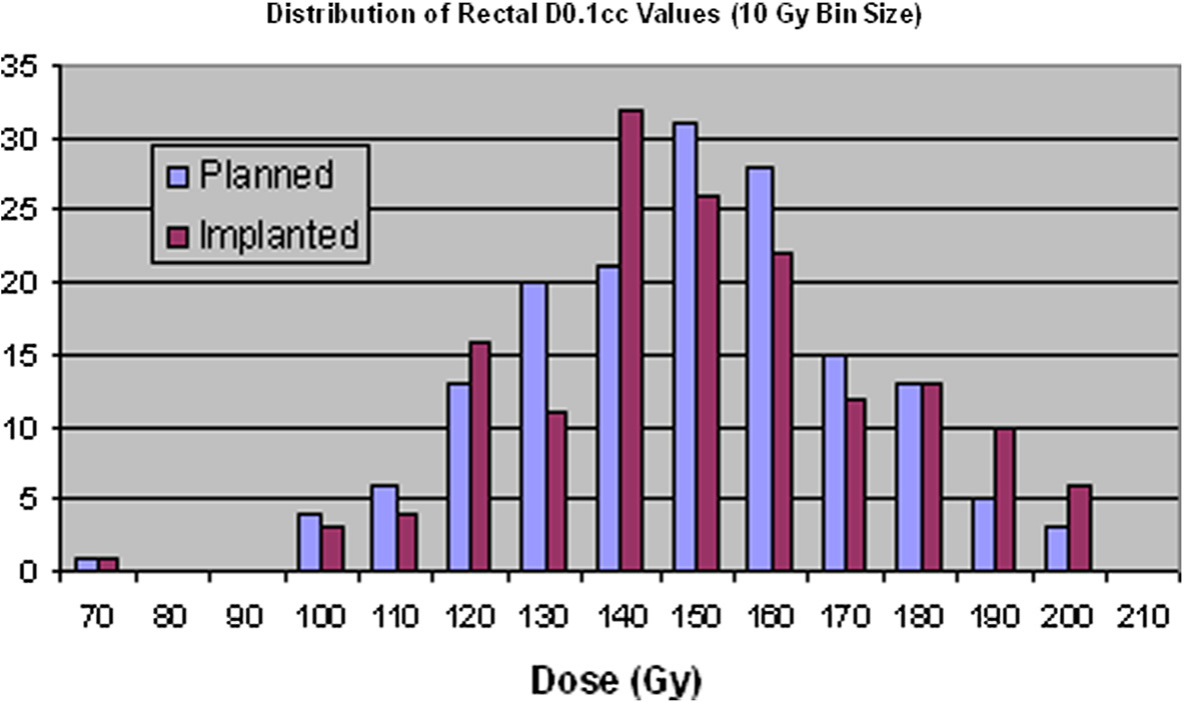
Distribution of rectal D0.1cc values for planned and implanted dosimetry.

The rectum dosimetry data given in Tables 7 and Table 8 show a statistically significant increase in the values for D0.1cc which is an indicator of the hot spot dose in the rectum. However, as with the urethra, it could be argued that the effect of this relatively small increase of only ~3% might be insignificant. In fact, compared to the dose distributions of the prostate and rectum, little clear change is visible in Figure 8 and Figure 9.

**Table 7:** Mean values for D2cc and D0.1cc for the rectum along with the associated standard deviations

**Dosimetry Parameter**	**Planned**	**St Dev**	**Implanted**	**St Dev**
**D2cc (Gy)**	77.7	18.6	80.0	22.6
**D0.1cc (Gy)**	145.3	26.6	149.6	30.0

**Table 8:** Percent differences and statistics for rectum data

**Dosimetry Parameter**	**Mean % Diff**	***t *test score**	**P value**
**D2cc**	3.07	2.42	0.0166
**D0.1cc**	3.00	2.91	0.0042

## Discussion

Needle misplacement is one common factor responsible for the loss of prostate coverage between planned and implanted dosimetry. This observation is confirmed by clinical studies reporting on source misplacements which ranged from 0.0 to 1.0 cm due to discrepancy between planned and achieved needle placement [9]. It is suggested that the adoption of real-time 3D computer planning system updates during I-125 implants significantly improves prostate coverage while reducing the doses to the organs at risk (the V150 for urethra of > 30% was reduced from 63% to 17% patients) [10].

As outlined earlier, there were insignificant (or very weakly significant) variations among the primary dosimetric parameters (D90 and V100) between planned and implanted dosimetry. This outcome is to be expected as D90 and V100 are the dosimetry parameters which are targeted in the planning process. This is especially evident with V100 which is not only targeted to be above 98% but has an upper bound of 100%. Thus it would not be expected to observe large deviations in this parameter unless the person implanting was completely missing the target.

Where significant change in dosimetric parameters is observed however, is in the ‘hotspot’ parameters (eg V150, V200) and OAR measures. The increase is indicative of a general ‘bunching’ of seeds towards clumps. This clumping could be due to the presence of pubic arch and other obstructions such as calcifications and already deployed seeds which will cause the path of the needle to be deflected. While each deviation of a needle may only be of the order of a millimetre, over a total implant of 60–90 seeds this could cause significant increases of hotspots, especially in the urethra which is generally located at the approximate centre of the prostate.

The most glaring example of increases in hotspots is typified by the elevated average V200 value of approximately 20% between planned and implanted dosimetry. It should be noted that extra seeds are implanted when needed at the end of the procedure to improve the dosimetry if need be. In the majority of cases this is not required, although in this study group 43% of patients did have extra seeds implanted. This was due to either: (1) making up for grossly miss placed seeds (mostly along superior/inferior direction due to poor seed deployment), i.e. more than 5mm from planned position, or (2) accounting for obvious differences in prostate shape (due to swelling as implant progresses) between planned volume and implanted volume. The benefit of real-time dose calculation updates enables the radiation oncologist to decide if the extra seeds were needed or not. The average number of extra seeds implanted was small, i.e. 2.6 (with a range of −1 to 12 extra seeds), and the most common number (i.e. more than 40% of cases) was 2 seeds. To put in perspective, the mean total number of seeds implanted per patient for the study group is 70 seeds. Therefore, the average 2.6 extra seeds added to 43% of the patients has a small influence on the overall study results (approximately < 2%), but is part of the real-time implant process and can not be discarded from analysis. It should also be noted that, in general, before extra seeds are implanted they are added to the real-time plan to see if they would be of benefit or not to the final dosimetry.

In the case of the OAR these increases in average doses are of the order of 3% which, while statistically significant, are likely to not be considered clinically important. It should be noted however that these levels of increases are clinic specific and influenced by the high level of experience in performing LDR seed implants by the urologists/oncologists in question. Thus for a new practice it is important that some level of live planning guidance be available in order to account for any errors in the implantation process.

The first work on real-time dosimetry was conducted on HDR brachytherapy for advanced prostate cancers [11,12]. LDR real-time dosimetry followed later, with one of the first reports being published by Stock and Stone (2000) [13] on a small cohort of 10 consecutive patients. Prada and colleagues [2] reported on a real-time dynamic intra-operative dose calculation technique which has replaced, in their practice, the need for post implant dosimetry. In order to avoid the challenges encountered during prostate delineation on post implant CT images, the group has implemented an interactive intra-operative dose calculation method whereby the treatment plan is designed on the spot according to anatomical specificities and limitations, with needles being positioned where considered most adequate, and not at given coordinates. The procedure is guided via TRUS and the needle positions are continuously updated on the Variseed 7.1 (Varian) planning system to reflect the real-time isodose distributions. The reported results showed good quality implants, with a median V90 of 98% and low normal tissue toxicity. Though our implant technique resembles to some extent to the one described above, post implant dosimetry remains to be part of the procedure in our centre.

While the aim of this project was to examine the differences in the planned dosimetry and the real-time updated implant dosimetry, there are more studies looking at dosimetric changes between the two-step procedure (volume study and planning followed weeks later by implant) and one-step live planning technique. Wilkinson et al. (2000) [14] have shown the improvements to dosimetry due to changing from the two-step to the one-step treatment technique via analysis of post implant dosimetry. The better dosimetric outcome with one-step technique can be attributed to the elimination of several uncertainties regarding patient setup and positioning, anatomical changes and organ movement. A comprehensive review on the intra-operative (real-time) planning techniques has analysed the advantages of real-time planning in overcoming the sources of errors in the two-step procedures mentioned above [15]. The real-time tracking of needles, as also confirmed by our study, was shown to offer a feedback on dosimetry, thus enabling immediate action for adjustments, when needed.

## Conclusions

This investigation assessed the accuracy of prostate seed implants by making comparisons between the planned dosimetry and actual implanted dosimetry. A general increase in the volume of high dose regions were the main differences observed for the implant dosimetry. This would be due to some needles being implanted closer together than planned. Hence, an increase in the size of hotspots within the prostate was observed, which did not necessarily have an effect on the size of the 145Gy isodose volume, as indicated by the small differences observed for V100.

Across the board increases in dose to OAR was also seen (in some cases significant), however the absolute changes in the OAR dosimetry were minimal and thus may not be of clinical concern.

The results show that geographical misses and/or needle displacements during implant may have repercussions for centres without real-time live planning due to the resultant dose distribution from the misplaced seeds not being taken into account.

The overall small changes observed confirms the precision of the implant technique which RAH has in place. However the ability to update the plan dosimetry with real-time planning, for the one step live planning technique, highlights that the implanted dosimetry can be improved.

## Abbreviations

LDR: Low Dose Rate; RAH: Royal Adelaide Hospital; I-125: Iodine 125; OAR: Organs At Risk; US: Ultrasound; TRUS: Trans Rectal Ultrasound; PTV: Planning Target Volume; PID: Post Implant Dosimetry; D90: Dose received by 90% of the volume; V100: Volume (in percent) which receives 100% of the prescribed dose; V150: Volume (in percent) which receives 150% of the prescribed dose; V200: Volume (in percent) which receives 200% of the prescribed dose; D10: Dose received by 10% of the volume; D30: Dose received by 30% of the volume; D0.1cc: Dose received by the hottest 0.1cc of the volume; D2cc: Dose received by the hottest 2 cc of the volume.

## Competing interests

The authors declare they have no competing interests.

## Authors’ contributions

JM carried out the data compilation and analysis as well as drafting the manuscript. LM provided the idea of the project, the review of the analysis as well as of the manuscript. JL provided overall approval of the research as well as revision of the analysis and manuscript. All authors read and approved the final manuscript.
